# Supplementary material for: Effect of Bioactive Packaging Materials Based on Sodium Alginate and Protein Hydrolysates on the Quality and Safety of Refrigerated Chicken Meat
Source: Polymers (Basel). 2024 Dec 6;16(23):3430. doi: 10.3390/polym16233430 (PMC11644077; doi:10.3390/polym16233430)
Supplement: Supplementary file 1 [file polymers-16-03430-s001.zip › polymers-3272947-supplementary.pdf]

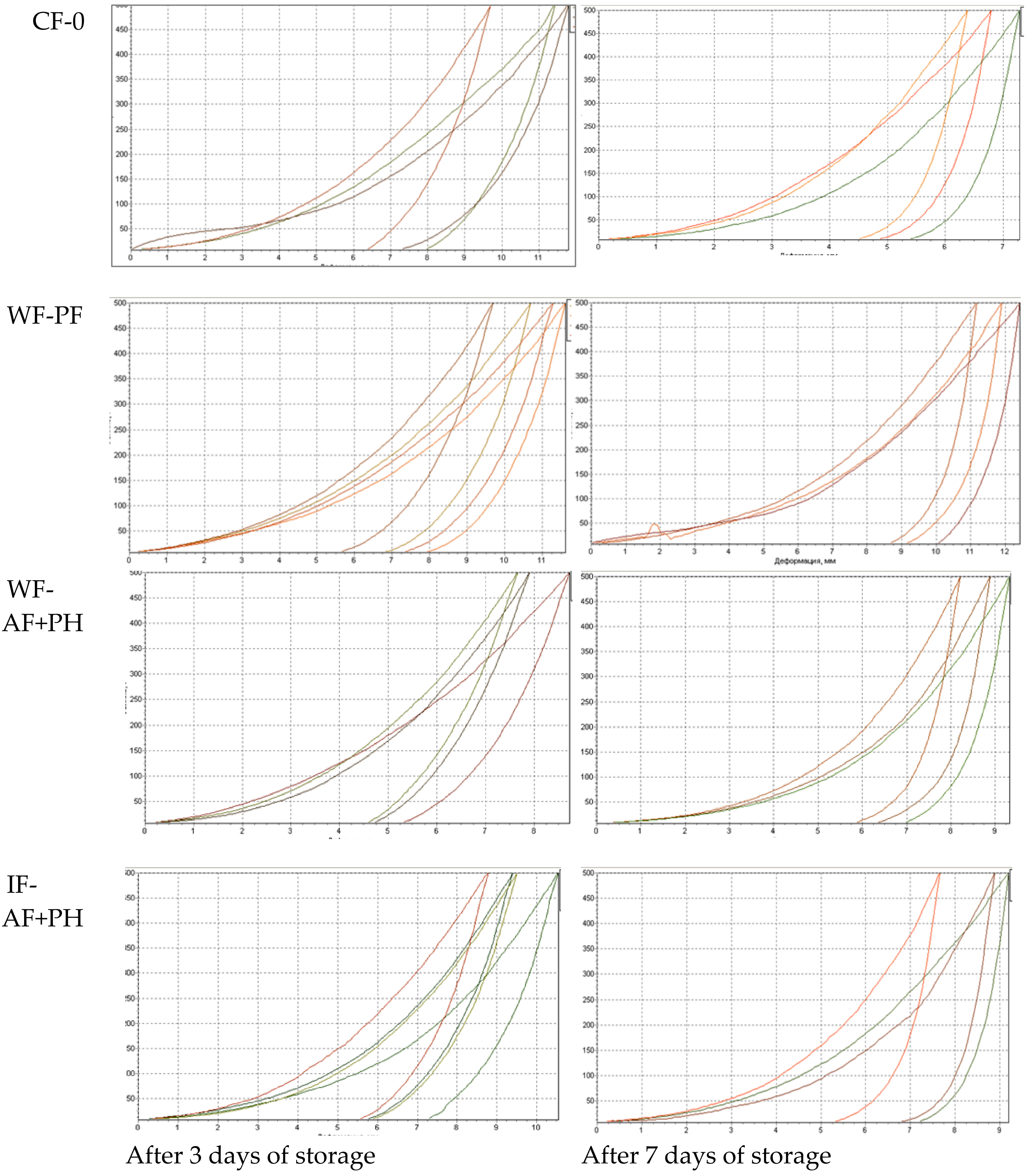

Figure S1. Characteristic type of mechanical stress relaxation curves of meat samples

Table S1. Change in chemical composition of poultry meat During storage

Storage period (Days)	CF-0	WF-PF	WF-AF	WF-AF+PH	IF-AF	IF-AF+PH
			Protein content, %			
0	22,83±0,21	22,64±0,18	22,91±0,16	22,90±0,20	22,78±0,19	22,69±0,12
3	23,27±0,14	22,51±0,13	23,68±0,23	23,09±0,13	23,18±0,16	23,07±0,23
7	23,91±0,25	21,33±0,09	24,12±0,14	23,87±0,19	23,69±0,22	23,72±0,21
			Fat content, %			
0	2,31±0,07	2,34±0,09	2,32±0,09	2,32±0,09	2,34±0,08	2,33±0,07
3	2,34±0,05	2,29±0,08	2,34±0,04	2,36±0,08	2,36±0,07	2,38±0,06
7	2,37±0,05	2,21±0,02	2,35±0,07	2,38±0,03	2,39±0,04	2,41±0,04

The data are the averaged values of triplicate repetition (±standard deviation); a, b, c, d—values with different letters in the same row differ at $p \leq 0.05$
